# The Australian Child Maltreatment Study (ACMS), a national survey of the prevalence of child maltreatment and its correlates: methodology

**DOI:** 10.5694/mja2.51869

**Published:** 2023-04-02

**Authors:** Divna M Haslam, David M Lawrence, Ben Mathews, Daryl J Higgins, Anna Hunt, James G Scott, Michael P Dunne, Holly E Erskine, Hannah J Thomas, David Finkelhor, Rosana Pacella, Franziska Meinck, Eva Malacova

**Affiliations:** ^1^ Queensland University of Technology Brisbane QLD; ^2^ Parenting and Family Support Centre, the University of Queensland Brisbane QLD; ^3^ Curtin University Perth WA; ^4^ Bloomberg School of Public Health Johns Hopkins University Baltimore MD United States of America; ^5^ Institute of Child Protection Studies Australian Catholic University Melbourne VIC; ^6^ Child Health Research Centre, the University of Queensland Brisbane QLD; ^7^ QIMR Berghofer Medical Research Institute Brisbane QLD; ^8^ Institute for Community Health Research Hue University Hue City Vietnam; ^9^ The University of Queensland Brisbane QLD; ^10^ Queensland Centre for Mental Health Research Brisbane QLD; ^11^ Crimes against Children Research Center University of New Hampshire Durham NH United States of America; ^12^ Institute for Lifecourse Development University of Greenwich London United Kingdom; ^13^ University of Edinburgh Edinburgh United Kingdom; ^14^ University of the Witwatersrand Johannesburg Johannesburg South Africa

**Keywords:** Child welfare, Child abuse, Epidemiology, Mental disorders, Healthcare disparities

## Abstract

**Objectives:**

To describe the aims, design, methodology, and respondent sample representativeness of the Australian Child Maltreatment Study (ACMS).

**Design, setting:**

Cross‐sectional, retrospective survey; computer‐assisted mobile telephone interviewing using random digit dialling (computer‐generated), Australia, 9 April – 11 October 2021.

**Participants:**

People aged 16 years or more. The target sample size was 8500 respondents: 3500 people aged 16–24 years and 1000 respondents each from five further age groups (25–34, 35–44, 45–54, 55–64, 65 years or more).

**Main outcome measures:**

Primary outcomes: Emotional abuse, neglect, physical abuse, sexual abuse, exposure to domestic violence during childhood, assessed with the Juvenile Victimization Questionnaire‐R2 Adapted Version (Australian Child Maltreatment Study). Secondary outcomes: selected mental disorder diagnoses (Mini International Neuropsychiatric Interview, MINI), selected physical health conditions, health risk behaviours, health service use.

**Results:**

The demographic characteristics of the ACMS sample were similar to those of the Australian population in 2016 with respect to gender, Indigenous status, region and remoteness category of residence, and marital status, but larger proportions of participants were born in Australia, lived in areas of higher socio‐economic status, had tertiary qualifications, and had income greater than $1250 per week. Population weights were derived to adjust for these differences. Associations between the number of calls required to recruit participants and maltreatment rates and health outcomes were not statistically significant.

**Conclusions:**

The ACMS provides the first reliable estimates of the prevalence of each type of child maltreatment in Australia. These estimates, and those of associated mental health and health risk behaviours reported in this supplement can inform policy and practice initiatives for reducing the prevalence of child maltreatment and its consequences. Our benchmark study also provides baseline data for repeated waves of the ACMS that will assess the effectiveness of these initiatives.

**The known**: Mistreatment during childhood has a range of negative effects throughout life. Reliable estimates of the prevalence of maltreatment in Australia have not been published.

**The new**: The Australian Child Maltreatment Study (ACMS) provides initial national data for estimating the prevalence of the five major types of child maltreatment and their consequences for mental and physical health across life.

**The implications**: Stratified sampling by age group allows changes over time and associations with health outcomes across life to be assessed. The ACMS delivers robust evidence for policy and practice with the aim of averting and ameliorating the consequences of mistreatment during childhood.

Mistreating children has negative effects during their childhood,^1^ across their life,^2^ and between generations.^3^ Reliable Australian data on the prevalence and nature of maltreatment by type are needed to inform policies and practices for protecting children. The Australian Royal Commission into Institutional Responses to Child Sexual Abuse called for studies of the prevalence of maltreatment over time in recommendation 2.1.^4^ In 2021, the Australian National Strategy to Prevent and Respond to Child Sexual Abuse was launched. The Australian government emphasised the need to improve the evidence base regarding maltreatment and committed itself to supporting further studies that track changes in prevalence attributable to the strategy.^5^

In this article, we describe the methodology of the Australian Child Maltreatment Study (ACMS). The ACMS is the first nationally representative study of the prevalence of child maltreatment and its correlates in Australia, and only three other nationally representative studies anywhere^6^, ^7^, ^8^ have assessed the five major forms of child maltreatment (emotional abuse, neglect, physical abuse, sexual abuse, exposure to domestic violence) throughout childhood to age 18 years. As there is no consensus about which experiences constitute child maltreatment, not all studies of its prevalence and outcomes have assessed the same types of maltreatment.^9^ Emotional abuse, neglect, physical abuse, and sexual abuse are broadly recognised as child maltreatment in policy, law, and practice around the world.^9^ Exposure to domestic violence is increasingly regarded as a distinct domain of child maltreatment with important adverse effects;^6^ it was included by twelve of the eighteen national prevalence studies in a 2020 systematic review.^9^ Further, the Juvenile Victimization Questionnaire (on which the ACMS instrument was based) measures exposure to domestic violence; this maltreatment type has been assessed by studies in nations comparable with Australia, such as the United Kingdom^10^ and Canada.^11^ Finally, our inclusion of all five maltreatment types in our instrument was endorsed during testing phases by our international technical expert panel.

The primary goals of the ACMS are to establish a robust benchmark dataset for estimating the prevalence of child maltreatment in Australia; to separately examine associations between maltreatment and maltreatment type with mental health, physical health, and health risk behaviours throughout life; and to estimate the burden of disease associated with child maltreatment. We expand on our study protocol^12^ by describing the design, measures, safety protocols, response rates, and sample representativeness in greater detail. This article is the methodological foundation for the other articles in this supplement^13^, ^14^, ^15^, ^16^, ^17^ and for other studies based on the dataset.

## Method

The ACMS is a cross‐sectional survey study of people in Australia aged 16 years or more about their childhood maltreatment experiences and health. The computer‐assisted telephone interview design allows survey items to be displayed or skipped according to participant responses. It also includes automatic prompts to check participant wellbeing and provide information about support at key stages of the interview or in response to specific answers. We oversampled young people (16–24 years) to provide a large baseline database with which the results of subsequent ACMS waves can be compared to assess changes in prevalence concomitant with policy, societal, and legal changes. The ACMS is conducted in accordance with the highest ethical and legal principles and scientific standards.^18^

### Sampling frame and participants

We used a mobile phone sampling frame and random digit dial methodology (computer‐generated numbers). In 2021, 99% of Australians used mobile phones.^19^ Respondents were eligible for participation if they were aged 16 years or more, and in an age group for which participants were required when contacted; they were excluded if their command of English was insufficient for participation. The target sample size was 8500 respondents: 3500 people aged 16–24 years and 1000 respondents from each of five further age groups (25–34, 35–44, 45–54, 55–64, 65 years or more). The sample size provides 80% power to estimate prevalence within one percentage point and to detect gender differences of 2.6 percentage points; it also provides 80% power to detect a two percentage point change in prevalence between studies.^12^

### Interviewer training and welfare

A professional survey company, the Social Research Centre (https://srcentre.com.au), conducted the computer‐assisted telephone interviews. Professional interviewers of various gender identification, age, and ethnic background with experience in conducting telephone interviews about sensitive topics administered the survey after comprehensive training. Training sessions, focused on the practical and logistic aspects of the study, were led by the lead author (DMH), a registered clinical psychologist, and Social Research Centre managers. A three‐stage training and support process optimised interviewer competence and ensured interviewer and participant welfare. First, we provided pre‐training material on the nature, scope, and significance of the study, and information about interviewers monitoring and responding to their own reactions. Second, we provided a 6‐hour online group training session on survey content, interviewee and interviewer responses, safety and distress protocols, self‐care, and general call management, using didactic, interactive, and role play methods. Finally, interviewers received ongoing support in online chats, supervisor debriefings, and group and personal employee assistance sessions; access to a clinical psychologist and psychiatrist was also provided. Instruction in the administration of the Mini‐International Neuropsychiatric Interview (MINI)^20^ used a train‐the‐trainer approach. The lead author and the first set of interviewers received formal training in administering the MINI by the owner of the instrument. The lead author provided training to others in subsequent training sessions. A computer‐assisted telephone interviewing version of the MINI provided interviewers with built‐in skip logic and scoring.

### Participant safety and welfare

We developed two protocols to ensure participant safety.^12^ First, we had a red flag protocol for protecting participants at current risk of harm from maltreatment. Participants aged 16 or 17 years who reported sexual or physical abuse during the preceding year were flagged for follow‐up, and a registered clinical psychologist phoned them to provide support, identify the nature and magnitude of any risk, and ensure their safety. Second, a comprehensive distress protocol responded to any participant distress, whereby the degree of response increased with distress level. Further details about these protocols will be reported in a separate publication.

### Data collection

The interviews were conducted during 9 April – 11 October 2021, the peak of the COVID‐19 pandemic and associated lockdowns in Australia, although Victoria was the only state with a sustained lockdown during this period. A text message was sent to each randomly generated phone number with information about the study and a link to the ACMS website (www.australianchildmaltreatmentstudy.org), which provided further survey and consent details, and options for opting into or out of participation. Those who opted out were not contacted again; those who opted in were phoned and invited to participate and to provide informed verbal consent. A maximum of eight calls were made to each phone number to recruit participants for the survey. All data will be deposited with the Australian Data Archive (https://ada.edu.au) in 2024 and will be accessible on application to the study authors.

### Measures and outcomes

The primary instrument in our study was the Juvenile Victimization Questionnaire‐R2: Adapted Version (Australian Child Maltreatment Study) (Box 1). Prior to the main survey, a multi‐stage process of instrument development, refinement, and pilot testing checked the performance and validity of the adapted items, including conceptual mapping of the original JVQ‐R2 maltreatment items to ensure congruence with conceptual models of child maltreatment; item modifications; expert review; cognitive testing; feedback from people who have experienced maltreatment, to assess face validity; and psychometric evaluation of pilot data. This process has been described elsewhere.^28^ Detailed information on maltreatment constructs and item wording is provided in the Supporting Information, tables 1 and 2.

Box 1 **Australian Child Maltreatment Study questionnaire modules and content**

**Table:** 

Content area	Measurement tool
Demographic information	Purpose‐designed items to match major Australian surveys. Gender and sexuality were assessed with the open‐ended questions “How would you define your gender?” and “How would you identify your sexuality?”. Responses were coded using a list of 14 response options. If required, interviewers read the first few options as a prompt. We conceptualised anyone who did not identify as male/man or female/women as being of diverse gender, and anyone who did not identify as being heterosexual has being of diverse sexuality.
Criminal justice system involvement	Purpose‐designed items that assessed how many times the participant had been arrested, convicted, or imprisoned.
Use of and attitude to corporal punishment	Purpose‐designed items assessing parental status, any use of corporal punishment as a parent or caregiver (yes/no), and frequency of use of corporal punishment.
Peer bullying and sibling victimisation	Purpose‐designed items based on the Olweus definition of peer bullying.^21^ Experiences and frequency of verbal, relational, and physical bullying are assessed. For verbal and relational bullying, we assessed whether it had been in person, online, or both. A final item about perceived reason for bullying provided options of race/ethnicity, sexuality or gender identity, and disability or impairment. Similar items were used to assess sibling verbal and physical victimisation.
Adverse childhood experiences	Adverse experiences not related to maltreatment were assessed using a subset of National Survey of Child Health items.^22^ Maltreatment‐related experiences were assessed using the Juvenile Victimization Questionnaire‐R2: Adapted Version (Australian Child Maltreatment Study).
Emotional abuse Neglect Physical abuse Sexual abuse Exposure to domestic violence Corporal punishment* Internet sexual victimisation (aged 16–24 years only)^†^	Juvenile Victimization Questionnaire‐R2: Adapted Version (Australian Child Maltreatment Study): dichotomous response questions (yes/no), with follow‐up items to capture contextual information: frequency of maltreatment (five major types); age at onset (five major types); age at cessation (five major types); relationship to persons who inflicted physical abuse, emotional abuse, sexual abuse; disclosure of physical abuse, sexual abuse (further details: Supporting Information, tables 1 and 2). Emotional abuse was assessed by three items, neglect by three items, physical abuse by two items, corporal punishment by one item, internet victimisation by two items, sexual abuse by five items (including one on sexual harassment), and exposure to domestic violence by four items.
Intimate partner violence	Intimate partner violence was assessed using a subset of the Composite Abuse Scale (Revised)‐Short Form (CAS_R_‐SF).^23^ Lifetime prevalence (by any partner) was assessed, but not frequency.
Mental disorders	Diagnoses of mental disorders — major depressive disorder (lifetime), post‐traumatic stress disorder (current), alcohol use disorder (current), generalised anxiety disorder (current) — were assessed with the Mini‐International Neuropsychiatric Interview (MINI).^20^
Suicide and non‐suicidal self‐injury	Suicide attempts and non‐suicidal self‐injury (for each: lifetime attempts, age at onset, attempts during preceding year) were assessed using items from the National Adolescent Mental Health Survey.^24^
Health risk behaviours and chronic health conditions	Tobacco use, obesity, and alcohol use were assessed using items from the National Survey of Mental Health and Wellbeing (2007).^25^ Cannabis dependence was assessed using the Severity of Dependence Scale.^26^ Chronic health conditions (diabetes or high sugar, stroke, heart disease) were assessed using items from the National Survey of Mental Health and Wellbeing (2007).^25^ Additional items were added to assess bipolar disorder diagnosis and sexually transmitted diseases.
Health service use	Health service use during the preceding year was assessed using a subset of items from the National Survey of Mental Health and Wellbeing 2007: overnight hospital admissions (number; length; reasons) and numbers of consultations with physical and mental health professionals.^25^
Final items, willingness to be recontacted	Final items assessed the presence and extent of survey‐related upset, based on items from the original Juvenile Victimization Questionnaire^8^ and the National Survey of Children's Exposure to Violence.^27^ These items were randomly used for 50% of participants. A single question assessed willingness to be contacted again for similar surveys.

* Corporal punishment was not deemed maltreatment, as it is not illegal in Australia and is not universally understood as physical abuse. Our conservative approach avoided inflating our estimate of physical abuse.

† Internet sexual victimisation items (grooming by adults, non‐consensual sharing of sexual images by anyone) were administered only to people aged 16–24 years who had internet access during childhood. Endorsements of these items were not included when estimating the overall prevalence of sexual abuse, but will be separately analysed, as will the sexual abuse item on harassment.

The primary outcomes were the proportions of respondents who reported physical abuse, sexual abuse, emotional abuse neglect or exposure to domestic violence during childhood. Secondary outcomes were selected mental disorders (using the MINI): major depressive disorder (lifetime), post‐traumatic stress disorder (current), generalised anxiety disorder (current), and alcohol use disorder (current); selected physical health conditions (diabetes or high blood sugar levels, heart disease, sexually transmitted infection); health risk behaviours and conditions (harmful drinking, cannabis dependence, suicide attempts, non‐suicidal self‐injury, tobacco use, obesity); and health service use.

### Statistical analysis

Response data are summarised as counts and proportions. The demographic characteristics of the ACMS sample were compared with those of the Australian population as determined by the 2016 national census,^29^ both directly and after weighting. Population weights were calculated using generalised raking^30^ to calibrate the survey sample to the estimated resident population of Australia aged 16 years or more on 30 June 2021, by gender, age group, Indigenous status, country of birth, highest education level, and Socio‐Economic Indexes for Areas (SEIFA) Index of Relative Socio‐economic Advantage and Disadvantage.^31^

### Quality assurance

Computer‐assisted telephone interviewing minimised interviewer error by automating skip and display patterns. Prior to the study, we tested display logic with dummy data and in test interviews. Interview administration was validated by listening to 5% of interviews to ensure that items were administered as intended and that interviewers followed protocols. Initial interviews by each interviewer were also monitored by survey company senior staff and academic project staff. Interviews were monitored for appropriate administration of all items and the application of all applicable protocols; all protocol deviations were discussed with the interviewer. We monitored data analyses in random spot checks of SAS 9.4 code and checking of analysis results in SPSS 27. Data code was checked by the statistical lead, or the study statistician if the statistical lead had conducted the analyses. The project manager repeated 30–50% of all primary analyses (for each article in this Supplement), randomly selected, as an additional check.

### Ethics approval

The Queensland University of Technology Human Research Ethics Committee approved the study (1900000477).

## Results

A total of 404 180 phone numbers were rung. No contact was made in 249 291 cases (61.7%); we estimated that 149 570 of these numbers were for people who would have been eligible and in an appropriate age group for our survey (60%; based on census fraction of households in each stratum). A further 15 563 numbers (3.8%) were for people we deemed ineligible on first contact (under 16 years of age or age quota already filled); 87 168 people refused participation before their eligibility could be determined (21.6%), of whom an estimated 52 300 would have been eligible (24.9%). In total, we estimated that 210 373 people would have been eligible to participate, and contact was made with 60 803 (28.9%) (Box 2).

Box 2 **Outcomes of phone calls to randomly generated mobile phone numbers**

**Table:** 

Call outcome	Phone numbers	Estimated in‐scope phone owners	Mean number of calls (SD)
Total calls made	404 180	210 373	2.2 (1.2)
No contact made	249 291 (61.7%)	149 570 (71.1%)	2.8 (0.8)
Contact made	154 889 (38.3%)	60 803 (28.9%)	1.4 (1.1)
Refused participation	87 168 (21.6%)	52 300 (24.9%)	1.3 (1.1)
Age quota filled	43 450 (10.8%)	0	1.7 (1.0)
Ineligible	15 768 (3.8%)	0	1.0 (1.0)
Completed interviews	8503 (2.1%)	8503 (4.0%)	2.1 (1.3)

SD = standard deviation.

Of 8773 people who commenced interviews, 8503 completed them (retention rate, 97.4%). The final response rate with respect to the estimated number of eligible candidates was 4.0%; with respect to eligible candidates contacted it was 14.0%. Most people who refused were too busy or had insufficient time to participate.

The mean interview time was 26.8 minutes (standard deviation, 8.1 minutes); the median length was 25.7 minutes (interquartile range, 21.3–31.0 minutes. Eighteen people who received initial text message invitations to participate submitted complaints (0.004%). Three study participants submitted complaints (0.04%); two were about interviewer style, one was related to the phrasing of the sexuality item. There were no complaints regarding the maltreatment or adversity survey items. No adverse outcomes were reported.

### Population representativeness of the respondent sample

The demographic characteristics of the ACMS sample were similar to those of the Australian population in 2016 (both with and without weighting) with respect to gender, Indigenous status, region and remoteness category of residence (Accessibility/Remoteness Index for Australia, ARIA+^32^), and marital status, but larger proportions of participants were born in Australia, lived in areas of higher socio‐economic status, had tertiary qualifications, or had income greater than $1250 per week. The intentionally higher proportion of participants aged 16–24 years had an impact on some characteristics; after adjusting for age, the ACMS sample and the Australian population were similar with respect to birthplace in Australia or overseas (self and parents) (Box 3).

Box 3 Demographic characteristics of the Australian Child Maltreatment Study (ACMS) respondent sample (2021), and of Australians aged 16 years or more (2016)*

**Table:** 

	Australian Child Maltreatment Study			2016 census
		Proportion		Proportion
	Number	Unweighted	Weighted^†^	
Gender (self‐identified)				
Men	4195	49.3%	48.1%	48.8%
Women	4182	49.2%	50.9%	51.2%
Non‐binary/other	126	1.5%	1.0%	NA^‡^
Age group (years)				
16–24	3500	41.1%	13.6%	13.6%
25–34	1000	11.8%	18.2%	18.2%
35–44	1000	11.8%	17.0%	17.0%
45–54	1002	11.8%	15.7%	15.7%
55–64	1001	11.8%	14.5%	14.5%
65 or more	1000	11.8%	20.9%	20.9%
Indigenous status				
Aboriginal or Torres Strait Islander	290	3.4%	2.7%	2.4%
Non‐Indigenous	8176	96.2%	96.8%	91.5%
Not stated	37	0.4%	0.5%	6.2%
Marital status				
Single/never married	4046	47.7%	29.8%	27.4%
Living together but not married	918	10.8%	11.0%	15.3%
Married	2715	32.0%	43.9%	42.7%
Separated/divorced/widowed	803	9.4%	15.3%	14.6%
Residence: region^33^				
Metropolitan	5798	68.2%	64.4%	67.0%
Regional/rural	2705	31.8%	35.6%	33.0%
Residence: remoteness^33^				
Major cities	6247	73.5%	69.6%	72.1%
Inner regional	1471	17.3%	19.1%	18.0%
Outer regional	658	7.7%	9.5%	8.1%
Remote	83	1.0%	1.2%	1.0%
Very remote	44	0.5%	0.6%	0.8%
Birthplace of participant				
Born in Australia	6347	74.6%	65.9%	64.6%
Born overseas	2146	25.3%	34.0%	28.3%
Not stated	10	0.1%	0.1%	7.1%
Birthplace of parents				
Both parents born in Australia	4362	51.3%	48.9%	45.5%
One parent born in Australia	1351	15.9%	10.6%	9.8%
Both parents born overseas	2762	32.5%	40.2%	37.5%
Not known	28	0.3%	0.3%	7.2%
Highest level of education				
Postgraduate degree	1100	12.9%	8.1%	8.0%
Undergraduate degree	1859	21.9%	17.9%	17.7%
College certificate/diploma	1385	16.3%	19.6%	19.4%
Year 12	2273	26.8%	20.6%	21.2%
Trade certificate	692	8.1%	13.5%	13.4%
Year 10	1091	12.8%	18.3%	18.1%
Year 9 or less	78	0.9%	2.1%	2.1%
Employment status				
Employed full‐time	3601	42.5%	43.1%	39.1%
Employed part‐time	2372	27.9%	21.3%	20.3%
Unemployed	724	8.5%	7.4%	4.5%
Not in the labour force	1779	21.0%	28.2%	36.0%
Index of Relative Socio‐economic Advantage and Disadvantage^31^				
Lowest quintile	1086	12.8%	15.6%	15.6%
2nd quintile	1180	13.9%	15.9%	15.9%
3rd quintile	1497	17.6%	19.0%	19.0%
4th quintile	1938	22.8%	20.8%	20.8%
Highest quintile	2802	33.0%	28.7%	28.7%
Individual income (weekly)				
Lower than $500	2316	27.2%	25.1%	45.6%
$500–1249	2158	25.4%	24.0%	32.0%
$1250 or more	2496	29.4%	32.6%	22.4%
Not stated	1533	18.0%	18.3%	NA^‡^

* Source: 2016 census of population and housing (using TableBuilder Basic).^29^ Characteristics by age group are provided in the Supporting Information, tables 3 to 6.

† Weighted by gender, age group, Indigenous status, country of birth, highest education level, and Socio‐Economic Indexes for Areas Index of Relative Socio‐economic Advantage and Disadvantage.

‡ Not an option in 2016 census.

Interviews were completed on the first call for 3334 participants (39.2%); 475 interviews (5.6%) required five or more calls. Associations between the number of calls required to recruit participants and maltreatment rates and health outcomes were not statistically significant, except that the prevalence of alcohol use disorders was higher among those who required more calls (*P* = 0.005) (Supporting Information, figures 1 to 4). The prevalence of health outcomes (general health, obesity, smoking, frequency of alcohol use) was similar to those reported by the 2017 Australian National Health Survey,^33^ but larger proportions of ACMS participants reported that they in fair or poor health or were obese, and the proportion who reported never drinking alcohol was smaller (Box 4).

Box 4 **Estimated prevalence of selected health indicators in the Australian Child Maltreatment Study (weighted data) and the National Health Survey 2017^33^**

**Table:** 

	Australian Child Maltreatment Study		National Health Survey 2017
	Number	Proportion	Proportion
General health			
Excellent	1330	14.6% (13.7–15.5%)	20.9% (20.1–21.8%)
Very good	2891	32.5% (31.2–33.7%)	35.5% (34.7–36.3%)
Good	2858	34.6% (33.3–35.9%)	28.8% (28.1–29.5%)
Fair	1105	13.6% (12.7–14.6%)	11.0% (10.4–11.6%)
Poor	319	4.6% (4.0–5.3%)	3.7% (3.4–4.1%)
Smoking status			
Ever smoked	3176	46.0% (44.6–47.3%)	43.5% (42.6–44.3%)
Daily smoker	831	11.8% (10.9–12.8%)	13.3% (12.8–13.7%)
Body mass index			
Underweight (< 18.5 kg/m^2^)	264	2.1% (1.8–2.5%)	2.3% (1.2–3.5%)
Healthy weight (18.5–25 kg/m^2^)	3599	37.5% (36.1–38.8%)	39.9% (38.6–41.4%)
Overweight (25–30 kg/m^2^)	2467	33.6% (32.3–34.9%)	34.0% (33.0–34.9%)
Obese (≥ 30 kg/m^2^)	1794	26.8% (25.5–28.0%)	23.8% (22.8–24.5%)
Not stated	379	—	—
Alcohol use frequency (preceding twelve months)			
Never	1343	17.5% (16.4–18.5%)	23.6% (23.2–24.0%)
Less than once a month	1811	20.8% (19.7–21.9%)	15.5% (14.8–16.2%)
One to three days per month	2121	21.0% (19.9–22.0%)	18.1% (16.9–19.3%)
One to four days per week	2326	27.7% (26.5–28.9%)	31.1% (29.5–32.7%)
Every day/nearly every day	902	13.1% (12.1–14.0%)	11.7% (10.7–12.7%)

### Missing data

Most participants provided responses to all questions, including those about maltreatment; the overall item non‐response rate was lower than 1%. Non‐response was greatest for items on sexual abuse (by item: 65–77 non‐responses, 0.8–0.9%) (Box 5). Given the low non‐response rate, we conservatively categorised people who declined an answer as not having experienced the relevant maltreatment type when estimating prevalence.

For 29 participants, data for some sexual abuse follow‐up items were initially missing because of a programming error. To retrieve the data without causing unnecessary distress, a clinical psychologist attempted to contact 23 participants who had consented to repeat contact and had not initially reported distress; the nineteen who could be contacted provided the information without distress or need for further support.

Box 5 **Missing survey data, by Juvenile Victimization Questionnaire‐R2 Adapted Version (Australian Child Maltreatment Study) item**

**Table:** 

**Maltreatment type/survey item**	**Missing responses**
Emotional abuse	
EA1. Hostile interaction/denigration	21 (0.2%)
EA2. Rejection	23 (0.3%)
EA3. Emotional unavailability	65 (0.8%)
Neglect	
NEG1. Environmental neglect	12 (0.1%)
NEG2. Nutritional/physical neglect	16 (0.2%)
NEG3. Medical neglect	24 (0.3%)
Physical abuse	
PA1. Severe physical abuse	20 (0.2%)
PA2. Moderate physical abuse	26 (0.3%)
Corporal punishment	
CP1. Corporal punishment	32 (0.4%)
Internet victimisation*	
IV1. Non‐consensual sharing of sexual images	10 (0.3%)
IV2. Internet grooming	9 (0.3%)
Sexual abuse	
SA1. Sexual harassment	65 (0.8%)
SA2. Abusive exposure	66 (0.8%)
SA3. Touching (excludes intercourse)	72 (0.8%)
SA4. Attempted intercourse	65 (0.8%)
SA5. Abusive intercourse	77 (0.9%)
Exposure to domestic violence	
EDV1. Physical violence between parents	21 (0.2%)
EDV2. Serious threats of domestic violence	31 (0.4%)
EDV3. Damage of property or pets	40 (0.5%)
EDV4. Intimidation or control	58 (0.7%)

* Respondents aged 16–24 years only.

### Participant safety and welfare

A total of 59 participants (15% of those age 16 or 17 years) were flagged for follow‐up and were assessed by a clinical psychologist to ensure safety. Based on information provided by participants, no cases warranted referral to mental health care professionals, child protection agencies, or the police. A total of 77 distress‐related call alerts (0.1% of all participants) were made, of which six were deemed significant and referred to the ACMS team for mental health review. None required intensive intervention, suggesting that distress was transitory.

## Discussion

The ACMS provides the first large, nationally representative dataset on the prevalence in Australia of each of the five major types of child maltreatment and their health and behavioural correlates. Moreover, our data, stratified by maltreatment type, can help identify which children are at greatest risk of specific forms of maltreatment, by whom, and at what ages. This knowledge is essential for the development of national evidence‐based prevention and intervention initiatives. Further, the ACMS gathers data by age group, providing information that will aid understanding of changes in maltreatment over time, a broad evidence base that can identify where progress has occurred and where efforts need to be intensified.

We found the evidence needed can be gathered in Australia with the methodology used by the ACMS, achieving the objectives of the national policy.^5^ The low levels of distress among ACMS participants, their willingness to be recontacted, and their comprehensive responses to a broad survey indicate strong support for this research among people who have or have not experienced maltreatment as children. The oversampling of young people (16–24 years old) provides sufficient power for detecting small changes in prevalence in further survey rounds, allowing evaluation of the impact of policy and practice initiatives.

The JVQ‐R2 Adapted Version (Australian Child Maltreatment Study) survey instrument was rigorously adapted and tested,^10^ enabling robust assessment of a broad range of maltreatment sub‐domains for the five maltreatment types, and it captured detailed information about their context. Our use of the MINI as a diagnostic measure of mental disorder was a further strength, as most studies apply only symptom scales.^6^ Finally, the assessment of risk factors such as adverse childhood experiences (parental separation or divorce, parental imprisonment, living with someone who had a mental illness, had suicidal thoughts, was severely depressed, or misused alcohol or drugs) and bullying by peers or siblings allows the specific influence of maltreatment to be determined while controlling for potentially confounding factors.

Survey studies are subject to response bias if there are systematic differences between those who participate and those who do not. What we can know about people who did not participate is limited, but our respondent sample was generally representative of the Australian population with respect to most major characteristics, both before and after weighting, indicating that random digit mobile phone dialling is effective in this respect. Our findings regarding several health‐related outcomes were similar to those reported by the 2017 National Health Survey.^33^ People who had experienced child maltreatment may have been more interested in participating in our survey, or they may choose not to participate if they do not wish to discuss it.^34^ If people with experience of mistreatment as children were more likely to participate, they might be easier to recruit than other participants; however, we found that associations between the number of calls required to recruit participants and maltreatment rates or health outcomes were not statistically significant.

As the ACMS employs a cross‐sectional retrospective design, it cannot establish causal associations. Longitudinal studies would expand our understanding of life course processes, the impact of support for people who have experienced maltreatment as children, and mechanisms of influence and resilience.

### Limitations

The response rate for our survey was very low. This limitation is consistent with the generally marked decline in telephone survey response rates;^35^ for example, response rates for telephone public opinion polls conducted by the American think tank, the Pew Research Center, dropped from 36% to 9% during 1997–2016.^36^ The proportions of Aboriginal and Torres Strait Islander people in our survey corresponded to the national proportion, but the small number may nevertheless preclude subgroup analyses. Similarly, the survey is adequately powered to estimate the overall prevalence of maltreatment, but is less able to detect very small changes in specific types of maltreatment types with low prevalence. We intentionally included participants aged 16 and 17 years to allow consideration of prevalence during the preceding year and to provide a baseline for future surveys; however, this may have led to underestimation of prevalence because further maltreatment before the age of 18 years will not be included. Recall bias was possible, as in all surveys of past experience. Our survey provided data for assessing birth cohort prevalence and associated health outcomes through life, but, as older participants may have less accurate recall of more distant events or may have reframed their perceptions of some events, differences between birth cohorts should be interpreted with caution. We did not assess the degree of child maltreatment experienced by participants. Finally, our survey was conducted during the COVID‐19 pandemic, when declines in mental health were reported around the world,^37^ perhaps amplifying apparent relationships between maltreatment and adverse outcomes. However, lockdowns in Australia had only a mildly greater impact on people with mental problems.^38^

### Conclusion

The ACMS provides the first reliable estimates of the prevalence of each type of child maltreatment in Australia, differences between age groups, and correlates throughout life. The prevalence estimates and associated mental health and health risk behaviours reported in other articles in this supplement can be used to inform policy and practice initiatives for reducing child maltreatment and its consequences. Our benchmark study also provides baseline data for repeated waves of the ACMS that will assess the effectiveness of these initiatives.

## Open access

Open access publishing facilitated by Queensland University of Technology, as part of the Wiley – Queensland University of Technology agreement via the Council of Australian University Librarians.

## Agency roles

The NHMRC funded the ACMS. The Australian Government provided supplementary funding for several specific questions. The researchers were independent of the funders.

## Data sharing statement

Under the registered data management plan, final datasets will be stored on the Australian Data Archive; access details will be published on the ACMS website (www.australianchildmaltreatmentstudy.org) in 2024. Under a multi‐institutional agreement, the survey instrument is the intellectual property of the research team. It will be made available under a Creative Commons licence after an embargo period.

## Competing interests

No relevant disclosures.

## Supporting information


**Supporting Information**.
