# Supplementary material for: The Australian Child Maltreatment Study (ACMS), a national survey of the prevalence of child maltreatment and its correlates: methodology
Source: Med J Aust. 2023 Apr 2;218(Suppl 6):S5–S12. doi: 10.5694/mja2.51869 (PMC10953333; doi:10.5694/mja2.51869)
Supplement: Supplementary file 1 — Supporting Information. [file MJA2-218-S5-s001.pdf]

MJA
The Medical Journal of Australia

Supporting Information

Supplementary methods and results

This appendix was part of the submitted manuscript and has been peer reviewed.
It is posted as supplied by the authors.

Appendix to: Haslam DM, Lawrence D, Mathews B, et al. The Australian Child Maltreatment Study (ACMS), a national survey of the prevalence of child maltreatment and its correlates: methodology. *Med J Aust* 2023; doi: 10.5694/mja2.51869.

Table 1. Additional information on assessment of child maltreatment with the Juvenile Victimization Questionnaire-R2: Adapted Version (Australian Child Maltreatment Study)

Physical abuse	Two dichotomous (yes/no) screeners were asked assessing moderate and severe physical abuse. Follow-up items obtained details about frequency (discrete numbers), relationship to individuals who inflicted the abuse, age of onset, and age of cessation. Young people aged 16–17 years were also asked about past year prevalence. Disclosure of physical abuse was assessed by asking participants whether they had ever disclosed the events (yes/no), if so to whom, at what age and if the participant felt supported by the person to whom they disclosed. Institutional physical abuse and disclosure of institutional physical abuse was assessed by repeating follow-up items only about the institutional abuse. This was triggered if the participant listed any institutional perpetrator in response to the perpetrator question. If a perpetrator in a religious organisation was reported, details were obtained about the type of religious organisation.
Sexual abuse	Five dichotomous (yes/no) screeners were asked assessing sexual harassment, abusive exposure, contact abuse short of intercourse, attempted forced intercourse, and forced intercourse. Follow-up items obtained details about frequency (discrete numbers), relationship to individuals who inflicted the abuse, age of onset, and age of cessation. Young people aged 16–17 years were also asked about past year prevalence. Disclosure of sexual abuse was assessed by asking participants whether they had ever disclosed the events (yes/no), if so to whom, at what age and if the participant felt supported by the person to whom they disclosed. Additionally, a single follow-up disclosure item was asked where disclosure was reported but not to an authority (i.e., the person reported they disclosed to a parent, friend or some other person who did not have a legal obligation to report it). This was designed to identify if the abuse was ever disclosed to someone with a legal obligation to report it. Institutional sexual abuse and disclosure of institutional sexual abuse was assessed by repeating follow-up items only about the institutional abuse. This was triggered if the participant listed any institutional perpetrator in response to the perpetrator question. If a perpetrator in a religious organisation was reported detailed were obtained about the type of religious organisation. Note: Sexual harassment was excluded from estimates of the prevalence of sexual abuse. Non-contact abuse is defined to include abusive exposure. The other three items are considered contact abuse.
Emotional abuse	Three dichotomous (yes/no) screeners were asked assessing hostile interaction or denigration, rejection, and emotional unavailability. Follow-up items obtained details about frequency (duration), relationship to individuals who inflicted the abuse, age of onset, and age of cessation. Young people aged 16–17 years were also asked about past year prevalence. By conceptual definition, emotional abuse is inflicted by caregivers in the home. Therefore, items about institutional maltreatment were not asked.
Neglect	Three dichotomous (yes/ no) screeners were asked assessing environmental neglect, nutritional/physical neglect, and medical neglect. Follow-up items obtained details about frequency (duration), age of onset, and age of cessation. Young people aged 16–17 years were also asked about past year prevalence. By conceptual definition, neglect is experienced in the home environment. Therefore, items about institutional neglect were not asked.
Exposure to domestic violence	Four dichotomous (yes/no) screeners assessing exposure to physical violence between parents, exposure to serious threats of domestic violence, exposure to damage of property or pets, exposure to intimidation or control. Follow-up items obtained details about frequency of exposure (discrete numbers), age of onset, and age of cessation. Young people aged 16–17 years were also asked about past year prevalence.
Corporal punishment	One dichotomous (yes/ no) screener was asked assessing corporal punishment (separate to physical abuse), by parents and adult caregivers. Follow-up items obtained details about frequency (discrete numbers), relationship to individuals who inflicted the corporal punishment, and age of cessation. Young people aged 16–17 years were also asked about past year prevalence.
Internet victimisation (16–24-year-old respondents only)	Two dichotomous (yes/no) screeners were asked assessing unwanted online sharing of sexual images by anyone, and online grooming by adults. Follow-up items obtained details about age of onset, age of cessation, frequency (discrete numbers), and relationship to individuals who did the acts. Disclosure of internet victimisation was assessed by asking participants whether they had ever disclosed the events (yes/no), if so to whom, at what age and if the participant felt supported by the person to whom they disclosed. Institutional internet victimisation was measured by repeating follow-up items only about the institutional abuse. This was triggered if the participant listed any institutional perpetrator in response to the perpetrator question. If a perpetrator in a religious organisation was reported, details were obtained about the type of religious organisation.

With the exception of emotional abuse and neglect, participants who responded ‘yes’ to any item were deemed to have experienced the relevant type of maltreatment. Conceptual definitions of neglect and emotional abuse require a repeated pattern of behaviour (over a period lasting weeks).

Table 2. Maltreatment, corporal punishment, and online sexual victimisation items as assessed with the Juvenile Victimization Questionnaire-R2: Adapted Version (Australian Child Maltreatment Study)

Type of maltreatment	Item number	Maltreatment subtype
**Emotional abuse (3 items)**		
Did any of your parents insult you, humiliate you, or call you hurtful names?	EA 1	Hostile interaction/denigration
Did any of your parents tell you they hated you, didn't love you, wished you were dead or had never been born?	EA 1	Rejection
Did any of your parents often ignore you, or not show you love and affection?	EA 3	Emotional unavailability
**Neglect (3 items)**		
Was your home often unsafe or unhealthy? For example, it had toilets or sinks that didn't work, rubbish piled up, and things like that?	NEG 1	Environmental neglect
Were you often not provided with regular meals, baths or showers, or clean clothes?	NEG 2	Nutritional/physical neglect
When you were sick or injured, did your parent ever fail to get you medical care or take care of you?	NEG 3	Medical neglect
**Physical abuse (2 items)**		
Did an adult ever beat you up, hit you on the head or face, choke you, or burn you?	PA 1	Severe physical abuse
Did an adult ever hit, punch, kick, or physically hurt you?	PA 2	Moderate physical abuse
**Corporal punishment* (1 item)**		
Did an adult ever smack you or physically punish you to discipline you for your misbehaviour?	CP 1	Corporal punishment
**Internet sexual victimisation (16-24 yr olds only) * (2 items)**		
Did anyone ever use the internet or a mobile phone to share sexual images of you without your consent?	IV 1	Non-consensual sharing of sexual images
Did an adult ever ask you over the internet or a mobile phone to talk about sex or send sexual images?	IV 2	Internet grooming
**Sexual abuse (5 items)**		
Did anyone ever say, write or do something sexual to you that was offensive or intimidating? *	SA 1	Sexual harassment*
Did anyone ever look at your private parts when they shouldn't have, or make you look at their private parts?	SA 2	Abusive exposure
Did anyone ever touch your private parts when they shouldn't have, or make you touch their private parts?	SA 3	Abusive touching (contact abuse short of intercourse
Did anyone ever try to force you to have sex, even if it didn't happen?	SA 4	Attempted intercourse
Did anyone ever force you to have sex?	SA 5	Abusive intercourse
**Exposure to domestic violence (4 items)**		
Did you ever see or hear one of your parents get pushed, hit, choked, or beaten up by your other parent or their partner?	EDV 1	Exposure to physical violence between parents
Did you ever see or hear one of your parents seriously threaten to hurt your other parent?	EDV 2	Exposure to serious threats of domestic
During an argument, did any of your parents ever damage any property or pets, punch the wall, or throw something?	EDV 3	Exposure to damage of property or pets
Did you ever see or hear one of your parents intimidate or control your other parent, either verbally, sexually, financially, or by isolating them from friends or family?	EDV 4	Exposure to intimidation or control

* Not included in calculations of child maltreatment, but reported separately.

Table 3. Demographic characteristics of the Australian Child Maltreatment Study (ACMS) respondent sample aged 16–24 years (2021; 3500 people), and of Australians aged 16–24 years (2016)*

	Australian Child Maltreatment Study			2016 census
		Proportion		
	Number	Unweighted	Weighted†	Proportion
Gender (self-identified)				
Men	1748	49.9%	49.1%	50.7%
Women	1662	47.5%	48.6%	49.3%
Non-binary/other	90	2.6%	2.3%	NA†
Indigenous status				
Aboriginal or Torres Strait Islander	167	4.8%	4.6%	4.2%
Non-Indigenous	3323	94.9%	95.2%	90.1%
Not stated	10	0.3	0.3%	5.7%
Marital status				
Single/never married	3020	86.3%	87.9%	89.0%
Living together but not married	347	9.9%	8.4%	8.3%
Married	113	3.4%	3.3%	2.4%
Separated/divorced/widowed	13	0.4%	0.4%	0.3%
Residence: region[3]				
Metropolitan	2507	71.6%	70.9%	70.3%
Regional/rural	993	28.4%	29.1%	29.7%
Residence: remoteness[3]				
Major cities	2695	77.0%	76.1%	75.5%
Inner regional	537	15.3%	15.9%	15.8%
Outer regional	226	6.5%	6.8%	7.0%
Remote/very remote	42	1.2%	1.2%	1.7%
Birthplace of participant				
Born in Australia	2792	79.8%	73.3%	73.5%
Born overseas	704	20.1%	26.5%	20.5%
Not stated	4	0.1%	0.2%	6.0%
Highest level of education				
Undergraduate degree or higher	712	20.3%	11.1%	10.6%
College certificate/diploma	448	12.8%	11.4%	4.8%
Year 12	1603	45.8%	29.6%	42.2%
Trade certificate	703	20.1%	3.0%	38.4%
Year 9 or less	34	1.0%	4%	4.1%
Employment status				
Employed full-time	1126	32.2%	26.8%	24.8%
Employed part-time	1444	41.3%	42.4%	31.9%
Unemployed	437	12.5%	13.4%	10.1%
Not in the labour force	493	14.1%	17.5%	33.2%
Index of Relative Socio-economic Advantage and Disadvantage[1]				
Lowest quintile	433	12.4%	14.1%	15.3%
2nd quintile	484	13.8%	15.1%	14.9%
3rd quintile	609	17.4%	18.3%	18.6%
4th quintile	797	22.8%	21.3%	21.7%
Highest quintile	1177	33.6%	31.1%	28.8%
Individual income (weekly)				
Lower than $500	1398	39.9%	45.3%	70.8%
$500–1249	1026	29.3%	25.1%	25.7%
$1250 or more	371	10.6%	7.8%	3.5%
Not stated	705	20.1%	21.8%	NA†

* Source: 2016 census of population and housing (using TableBuilder Basic).[2]

† Not an option in 2016 census.

‡ Accessibility and Remoteness Index of Australia (ARIA+).[3]

Table 4. Demographic characteristics of the Australian Child Maltreatment Study (ACMS) respondent sample aged 25–44 years (2021; 2000 people), and of Australians aged 25–44 years (2016)*

	Australian Child Maltreatment Study			2016 census
		Proportion		
	Number	Unweighted	Weighted†	Proportion
Gender (self-identified)				
Men	992	49.6%	49.7%	49.3%
Women	986	49.3%	49.3%	50.7%
Non-binary/other	22	1.1%	1.1%	NA†
Indigenous status				
Aboriginal or Torres Strait Islander	66	3.3%	3.4%	2.5%
Non-Indigenous	1924	96.2%	96.2%	91.3%
Not stated	10	0.5%	0.5%	6.2%
Marital status				
Single/never married	666	33.3%	33.7%	29.0%
Living together but not married	363	18.2%	18.9%	20.4%
Married	853	42.7%	41.0%	44.7%
Separated/divorced/widowed	118	5.9%	6.4%	6.0%
Residence: region[3]				
Metropolitan	1482	74.1%	71.9%	72.0%
Regional/rural	518	25.9%	28.1%	28.0%
Residence: remoteness[3]				
Major cities	1581	79.1%	76.8%	76.6%
Inner regional	257	12.9%	13.5%	14.4%
Outer regional	133	6.7%	7.9%	6.9%
Remote/very remote	29	1.4%	1.8%	2.0%
Birthplace of participant				
Born in Australia	1324	66.2%	61.3%	60.0%
Born overseas	674	33.7%	38.8%	33.0%
Not stated	2	0.1%	0.1%	7.0%
Highest level of education				
Undergraduate degree or higher	1093	54.7%	38.1%	38.1%
College certificate/diploma	366	18.3%	21.8%	11.8%
Year 12	253	12.7%	16.8%	16.6%
Trade certificate or year 10	283	14.2%	22.6%	30.3%
Year 9 or less	5	0.3%	0.7%	3.1%
Employment status				
Employed full-time	1290	64.5%	61.8%	56.1%
Employed part-time	430	21.5%	21.8%	21.7%
Unemployed	145	7.3%	8.5%	5.0%
Not in the labour force	135	6.7%	7.9%	17.1%
Index of Relative Socio-economic Advantage and Disadvantage[1]				
Lowest quintile	234	11.7%	14.3%	13.8%
2nd quintile	225	11.3%	13.2%	14.2%
3rd quintile	340	17.0%	18.1%	18.9%
4th quintile	492	24.6%	22.8%	22.0%
Highest quintile	709	35.5%	31.6%	30.8%
Individual income (weekly)				
Lower than $500	257	12.8%	14.2%	31.2%
$500–1249	472	23.6%	26.1%	36.8%
$1250 or more	1,003	50.2%	45.4%	32.0%
Not stated	268	13.4%	14.3%	NA†

* Source: 2016 census of population and housing (using TableBuilder Basic).[2]
† Not an option in 2016 census.

Table 5. Demographic characteristics of the Australian Child Maltreatment Study (ACMS) respondent sample aged 45–64 years (2021; 2003 people), and of Australians aged 45–64 years (2016)*

	Australian Child Maltreatment Study			2016 census
		Proportion		
	Number	Unweighted	Weighted†	Proportion
Gender (self-identified)				
Men	964	48.1%	48.6%	48.7%
Women	1030	51.4%	51.0%	51.3%
Non-binary/other	9	0.4%	0.5%	NA†
Indigenous status				
Aboriginal or Torres Strait Islander	50	2.5%	2.5%	2.0%
Non-Indigenous	1943	97.0%	97.1%	92.0%
Not stated	10	0.5%	0.5%	6.0%
Marital status				
Single/never married	290	14.5%	15.1%	11.0%
Living together but not married	164	8.2%	7.7%	15.2%
Married	1185	59.2%	58.2%	59.4%
Separated/divorced/widowed	364	18.2%	18.9%	18.6%
Residence: region[3]				
Metropolitan	1251	62.5%	60.1%	64.3%
Regional/rural	752	37.5%	39.8%	35.7%
Residence: remoteness[3]				
Major cities	1363	68.1%	65.5%	69.2%
Inner regional	411	20.5%	21.8%	9.8%
Outer regional	188	9.4%	10.6%	9.0%
Remote/very remote	41	2.0%	2.2%	2.0%
Birthplace of participant				
Born in Australia	1500	74.9%	68.5%	61.9%
Born overseas	500	25.0%	31.4%	30.7%
Not stated	3	0.1%	0.1%	7.4%
Highest level of education				
Undergraduate degree or higher	820	40.9%	23.9%	25.0%
College certificate/diploma	412	20.6%	22.6%	12.3%
Year 12	282	14.1%	17.3%	13.0%
Trade certificate or year 10	471	23.5%	34.2%	41.5%
Year 9 or less	18	0.9%	2.0%	8.3%
Employment status				
Employed full-time	267	13.3%	16.2%	16.0%
Employed part-time	287	14.3%	16.3%	16.5%
Unemployed	350	17.5%	19.0%	19.3%
Not in the labour force	446	22.3%	20.0%	20.1%
Index of Relative Socio-economic Advantage and Disadvantage[1]				
Lowest quintile	234	11.7%	14.3%	13.8%
2nd quintile	225	11.3%	13.2%	14.2%
3rd quintile	340	17.0%	18.1%	18.9%
4th quintile	492	24.6%	22.8%	22.0%
Highest quintile	653	32.6%	28.5%	27.8%
Individual income (weekly)				
Lower than $500	306	15.3%	17.9%	36.4%
$500–1249	423	21.1%	22.9%	33.6%
$1250 or more	933	46.6%	41.2%	30.0%
Not stated	341	17.0%	18.0%	NA†

* Source: 2016 census of population and housing (using TableBuilder Basic).[2]

† Not an option in 2016 census.

Table 6. Demographic characteristics of the Australian Child Maltreatment Study (ACMS) respondent sample aged 65 years or more (2021; 1000 people), and of Australians aged 65 years or more (2016)*

	Australian Child Maltreatment Study			2016 census
		Proportion		
	Number	Unweighted	Weighted†	Proportion
Gender (self-identified)				
Men	491	49.1%	44.4%	46.4%
Women	504	50.4%	55.1%	53.6%
Non-binary/other	5	0.5%	0.5%	NA†
Indigenous status				
Aboriginal or Torres Strait Islander	7	0.7%	0.7%	0.8%
Non-Indigenous	986	98.6%	98.6%	92.2%
Not stated	7	0.7%	0.7%	7.0%
Marital status				
Single/never married	70	7.0%	6.4%	4.6%
Living together but not married	44	4.4%	4.2%	11.5%
Married	578	57.8%	54.9%	49.2%
Separated/divorced/widowed	308	30.8%	34.6%	34.8%
Residence: region[3]				
Metropolitan	1482	74.1%	71.9%	72.0%
Regional/rural	518	25. %9	28.1%	28.0%
Residence: remoteness[3]				
Major cities	608	60.8%	59.1%	66.0%
Inner regional	266	26.6%	26.6%	23.3%
Outer regional	111	11.1%	12.3%	9.5%
Remote/very remote	15	1.5%	2.0%	1.2%
Birthplace of participant				
Born in Australia	731	73.1%	65.0%	57.4%
Born overseas	268	26.8%	34.9%	33.4%
Not stated	1	0.1%	0.1%	9.2%
Highest level of education				
Undergraduate degree or higher	334	33.4%	17.0%	14.5%
College certificate/diploma	159	15.9%	16.1%	8.8%
Year 12	160	16.0%	18.8%	11.4%
Trade certificate or year 10	326	32.6%	43.9%	38.1%
Year 9 or less	21	2.1%	4.2%	27.1%
Employment status				
Employed full-time	98	9.8%	8.6%	5.5%
Employed part-time	91	9.1%	8.0%	6.9%
Unemployed	11	1.1%	1.0%	0.4%
Not in the labour force	800	80.0%	82.4%	87.2%
Index of Relative Socio-economic Advantage and Disadvantage[1]				
Lowest quintile	152	15.2%	18.0%	18.4%
2nd quintile	184	18.4%	20.5%	18.5%
3rd quintile	198	19.8%	21.0%	18.8%
4th quintile	203	20.3%	18.1%	18.7%
Highest quintile	263	26.3%	22.4%	25.5%
Individual income (weekly)				
Lower than $500	355	35.5%	40.5%	67.1%
$500–1249	237	23.7%	21.2%	25.4%
$1250 or more	189	18.9%	14.7%	7.5%
Not stated	219	21. 9%	23.6%	NA†

* Source: 2016 census of population and housing (using TableBuilder Basic).[2]

† Not an option in 2016 census.

Figure 1. Distribution of number of calls made to complete interviews

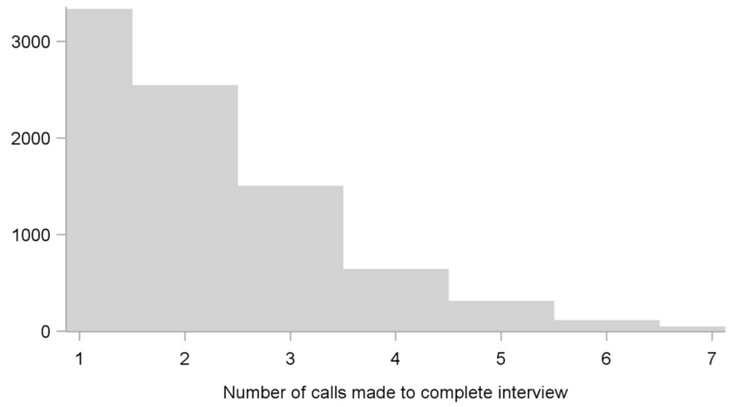

Figure 2. Relationship between number of calls made to complete the interview and prevalence of overall child maltreatment*

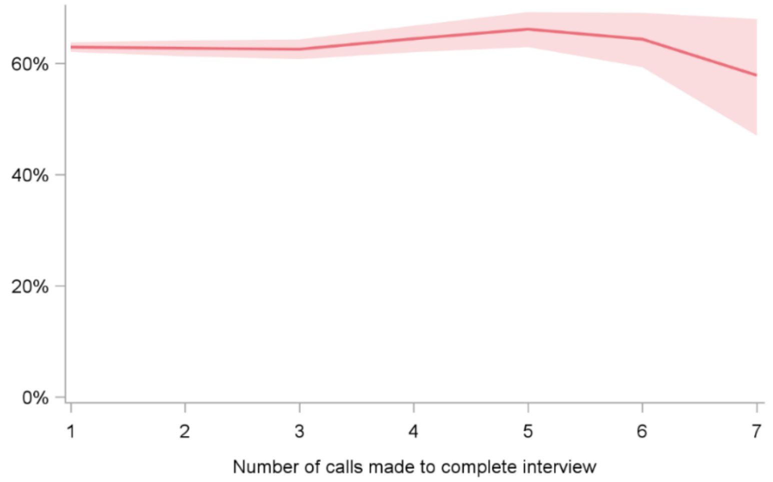

* We tested for a relationship between number of calls made to complete the interview and prevalence of child maltreatment using the framework of generalised additive models (GAMs), which allows for testing of both linear and non-linear relationships.[4] GAMs were used to fit a non-linear spline curve to the relationship between number of calls made and prevalence of child maltreatment and mental health outcomes. GAMs permit the data to inform the best fitting shape to the relationship between two variables, rather than impose a linear or other pre-defined form. There was no significant linear (*P* = 0.51) or non-linear (*P* = 0.24) relationship.

Figure 3. Relationship between number of calls required to complete the interview and prevalence of each of the five types of child maltreatment*

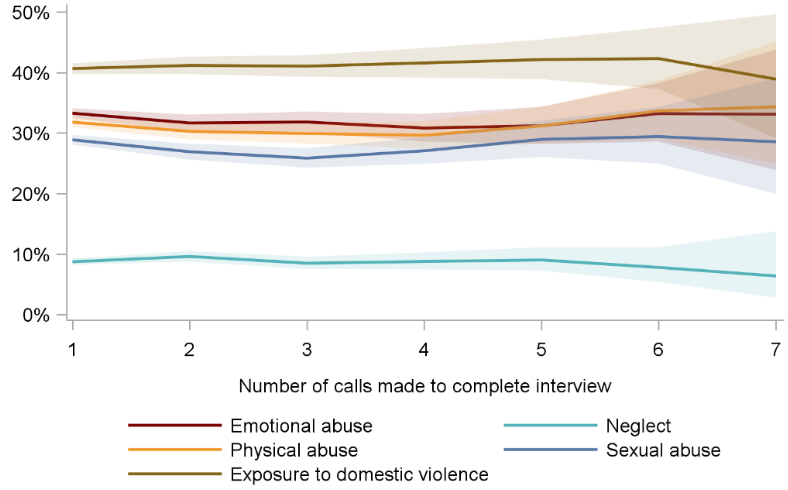

* Method: as for figure 2. No significant linear or non-linear relationship was found for any of the five types of maltreatment.

Figure 4. Prevalence of mental health conditions, by number of calls made to complete the interview*

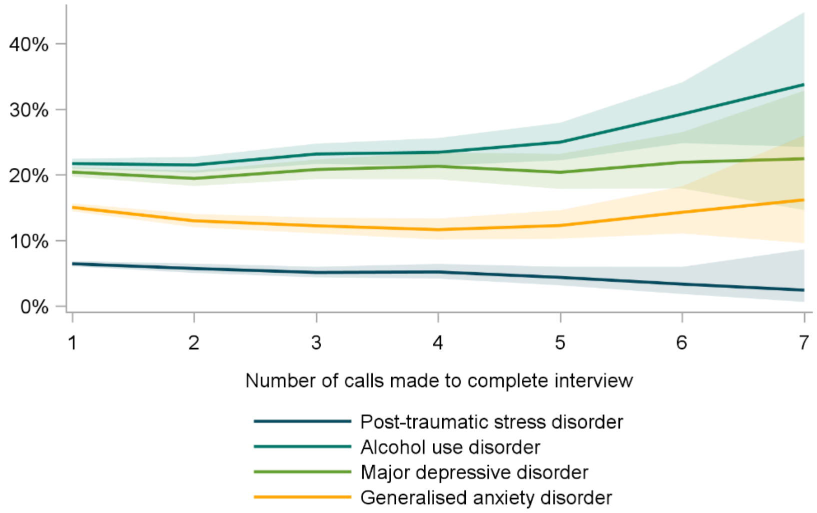

* Method: as for figure 2. The prevalence of alcohol use disorders was higher among those who required more calls to complete the interview ($P = 0.005$). Other changes were not statistically significant. People with alcohol use disorders may be more difficult to engage in telephone surveys and may take more effort to recruit.[5,6] Current level of functional impairment from mental illness, particularly associated with substance use, may be associated with participation in health surveys.

References

1. Australian Bureau of Statistics. 2033.0.55.001. Census of population and housing: Socio-Economic Indexes for Areas (SEIFA), Australia, 2016. IRSAD. 27 Mar 2018. https://www.abs.gov.au/ausstats/abs@.nsf/Lookup/by%20Subject/2033.0.55.001~2016~Main%20Features~IRSAD~20 (viewed Jan 2022).
2. Australian Bureau of Statistics. 2072.0. Census of population and housing: TableBuilder Basic, Australia, 2016. 4 July 2017. https://www.abs.gov.au/AUSSTATS/abs@.nsf/Lookup/2072.0Main%2BFeatures12016?OpenDocument (viewed Apr 2022).
3. Glover JD, Tennant SK. Remote areas statistical geography in Australia: notes on the Accessibility/Remoteness Index for Australia (ARIA+ version). Adelaide: Public Health Information Development Unit, University of Adelaide, 2003. https://digital.library.adelaide.edu.au/dspace/handle/2440/45471 (viewed Apr 2022).
4. Hastie TJ, Tibshirani RJ. Generalized additive models. New York: Routledge, 1990.
5. Maclennan B, Kypri K, Langley J, Room R. Non-response bias in a community survey of drinking, alcohol-related experiences and public opinion on alcohol policy. Drug and Alcohol Depend 2012; 126: 189-194.
6. Mieklejohn J, Connor J, Kypri K. The effect of low survey response rates on estimates of alcohol consumption in a general population survey. PLoS One 2012; 7: e35527.
